# Deep Vein Thrombosis Following Non-myeloablative Allogeneic Stem Cell Transplantation: Presentation of Three Cases and Literature Review

**DOI:** 10.4274/Tjh.92499

**Published:** 2013-06-05

**Authors:** Evren Özdemir, Emin Kansu

**Affiliations:** 1 Hacettepe University, Institute of Oncology, Stem Cell Transplantation Unit, Ankara, Turkey

**Keywords:** DVT, Non-myeloablative allogeneic stem cell transplantation, incidence

## Abstract

The incidence of deep vein thrombosis (DVT) after non-myeloablative (NMA) allogeneic stem cell transplantation (allo-SCT) is unknown. In addition, very few studies on the predisposing factors for DVT post SCT have been published. The incidence of DVT among patients that underwent NMA allo-SCT at our hospital was 4.1% (3 of 73) over the course of last 8 years, and to the best of our knowledge this is the first study to report the incidence of DVT following NMA allo-SCT. The present findings show that NMA allo-SCT patients may have multiple risk factors for DVT. Herein we present 3 cases of DVT following NMA allo-SCT and a literature review.

**Conflict of interest:**None declared.

## Abstract (tr)

Miyeloablatif olmayan allojeneik kök hücre nakli (NST) sonrası derin ven trombozu (DVT) sıklığı bilinmemektedir. Ek olarak, allojeneik nakil sonrası DVT gelişiminin nedenleri ile ilgili çok az sayıda çalışma mevcut olup, bu çalışmalarda izole bazı etiyolojik faktörler tanımlanmıştır. Merkezimizde NST protokolleri ile tedavi edilen hastalarda DVT gelişme sıklığı son sekiz yıl içinde %4,1 (73 hastada 3 vaka) olarak bulunmuş olup, bu hasta grubunda bildirilen ilk rapordur. NST sonrası DVT gelişimi ile ilgili gözlemimiz, bu hastalarda birden çok risk faktörünün bir arada bulunduğudur. Burada, bu hasta grubunda DVT gelişen üç vaka sunulmuş ve ilgili literatür gözden geçirilmiştir.

## INTRODUCTION

Deep vein thrombosis (DVT) increases the incidence of morbidity and mortality following myeloablative stem cell transplantation (SCT) [1,2,3,4,5,6,7]. The incidence of thromboembolic events following conventional myeloablative autologous and allogeneic (allo) SCT are approximately 5% and 15%, rrespectively [1]; however, the incidence of DVT following non-myeloablative (NMA) allo-SCT is unknown. In addition, very few studies on the predisposing factors for DVT post- SCT have been published. To the best of our knowledge the present study is the first to report the incidence of DVT following NMA allo-SCT. Herein we present 3 cases of DVT following NMA allo-SCT and a review of the literature. Written informed consents to publish their data were obtained from all 3 patients.

## CASE REPORTS

**Case 1**

A 47-year-old male underwent NMA allo-SCT (using fludarabine and total body irradiation (TBI) [200 cGy]) from an HLA-identical sibling due to high-risk acute myeloblastic leukemia (AML). His post-transplant course was complicated by chronic graft-versus host-disease (GVHD) of the skin (sclerodermatous type) and oral mucosa, which responded well to mycophenolate mofetil, systemic steroids, and photopheresis. The patient developed DVT of the left femoral vein and subsequent pulmonary emboli 5 months post transplantation while receiving the above-mentioned immunosuppressive treatments. The patient did not have a history of DVT; however, his family history included close relatives that died at early ages due to myocardial infarction and cerebrovascular events. As such, thrombophilia screening was performed, which showed factor (F) V Leiden homozygous mutation; this finding was confirmed by testing DNA obtained via oral mucosal swabbing. The patient was started on anticoagulation treatment with low molecular weight heparin (LMWH) with subsequent resolution of the DVT. The patient had long-term chronic GVHD necessitating low-dose steroid treatment. Life-long anticoagulation treatment with warfarin was planned.

**Case 2**

A 37-year-old male underwent NMA allo-SCT (using fludarabine and TBI [200 cGy]) from an HLA-identical sibling due to high risk AML. His post-transplant course was complicated by grade II acute GVHD of the gastrointestinal tract, followed by chronic GVHD of the oral mucosa. The patient responded well to treatment with mycophenolate mofetil and systemic steroids. He developed DVT of the left femoral vein 7 months post transplantation while the steroid treatment was being tapered. The patient had no history of DVT; however, as he was only 37 years old thrombophilia screening was performed, which showed a slightly low free protein S antigen level (45%) (normal range: 60%-130%). This finding was confirmed via subsequent confirmatory testing and family studies. The patient was given anticoagulation treatment with LMWH with subsequent resolution of the DVT. The patient’s chronic GVHD was successfully treated and steroids were completely tapered off. Nonetheless, the patient’s low free protein S antigen level persisted, suggesting hereditary deficiency; therefore, life-long anticoagulation treatment with warfarin was planned.

**Case 3**

A 40-year-old female underwent NMA allo-SCT (using fludarabine and TBI [200 cGy]) from an HLA-identical sibling due to secondary AML. One week post transplantation her left jugular catheter site was extremely painful and swollen. The catheter was withdrawn and doppler ultrasound confirmed a left jugular vein thrombus. She had no history of DVT, but because she was only 40 years old thrombophilia screening was performed, which showed low-level protein C activity (55%) (range: 70%-130%) and an elevated fibrinogen level (658 mg/dL). The DVT was treated with low molecular weight heparin. Protein C activity and the fibrinogen level returned to normal 30 d post transplantation. The patient was given anticoagulation treatment for 3 months only.

## DISCUSSION

SCT recipients have a high incidence of DVT. DVT has been reported to occur following conventional myeloablative autologous SCT and allo-SCT; however, little is known about the incidence of and risk factors for DVT following NMA allo-SCT. Patients undergoing SCT have known risk factors for the development of DVT, which include underlying malignancy, conditioning regimen, immobility during hospitalization, and the universal use of central venous catheters. In addition, there are several reports detailing the risk factors for the development of DVT post SCT. Decreases in circulating natural anticoagulants, protein C [2,3,4,5,6], protein S [3], ve AT-III [5] and an increase in fibrinogen [5] have been shown to occur following SCT, which cause a hypercoagulable state, a risk factor associated with thromboembolic events post transplantation. 

A large-scale study evaluated hemostatic complications in autologous SCT and allo-SCT patients, and reported that thromboembolic events were more common in allo-SCT recipients (15%) [1]. Chronic GVHD and treatment with steroids were the major factors associated with the occurrence of DVT [1]. Antiphospholipid syndrome with elevated anticardiolipin antibody titers, commonly in association with GVHD, has been reported to cause catastrophic thromboembolism following allo-SCT [6]. Another large-scale study reported that the incidence of DVT among allo-SCT recipients was about 5%; however, most of the patients received peri-transplant low molecular weight heparin prophylaxis. Additionally, the most frequent factor associated with DVT was catheter use, followed by GVHD [7]. FV Leiden is associated with a 3-8-fold increase in the risk of venous thrombosis in heterozygous patients, versus an 80-fold increase in homozygous patients [8]. Patients with FV Leiden mutation have a significantly higher risk for the development of DVT associated with other predisposing factors, especially with catheter insertion [8,9,10]. 

Previously reported risk factors for the development of DVT post SCT are summarized in Table 1. 

Most of our NMA allo-SCT patients had a diagnosis of lymphoid malignancy (n=28), followed by myeloid malignancy (n=22), multiple myeloma (n=14), and solid tumors (n=6). The NMA regimens used at our hospital were as follows: fludarabine+TBI (n=32, 46%); fludarabine+MEL (n=20, 28.5%); fludarabine+CTX (n=6, 8.5%); FLAG (n=6, 8.5%); fludarabine+BU (n=6, 8.5%). 

The incidence of DVT in the patients that underwent NMA allo-SCT at our hospital was 4.1% (3 of 73 cases). To the best of our knowledge the present study is the first to report the incidence of DVT following NMA allo-SCT. Although the number of patients was small, the incidence rate is lower than previously reported for conventional allo-SCT. None of the presented patients received peri-transplant DVT prophylaxis. Interestingly, all 3 patients with DVT were diagnosed as AML and received the fludarabine+TBI NMA regimen. It remains unclear if the diagnosis of AML and/or the conditioning regimen had any effect on the occurrence of DVT; however, this warrants further prospective studies. 

We observed that the 3 patients that developed DVT following NMA allo-SCT may have had multiple predisposing factors. Our patients had combinations of previously reported risk factors, further increasing the likelihood of DVT, as follows: Case 1: FV Leiden homozygous mutation, chronic GVHD, and steroid use; Case 2: Protein S deficiency, chronic GVHD, and steroid use; Case 3: low protein C activity, elevated fibrinogen, and catheter use. Case 1 had a strong family history, cases 2 and 3 belonged to a younger age group to justify thrombophilia screening. In cases 1 and 2 hereditary risk factors were noted as underlying the development of DVT; however, other acquired risk factors also likely added to the existing risk. Case 3 had no known hereditary risk factor; however, the acquired temporary risks were sufficient to cause DVT.

In conclusion, DVT following NMA allo-SCT may be associated with multiple predisposing factors with additive risks. Additional large-scale prospective studies are necessary to determine the precise incidence, at-risk populations, and the potential risks and benefits of thromboprophylaxis in this patient population.

## CONFLICT OF INTEREST STATEMENT

The authors of this paper have no conflicts of interest, including specific financial interests, relationships, and/or affiliations relevant to the subject matter or materials included. 

## Figures and Tables

**Table 1:**
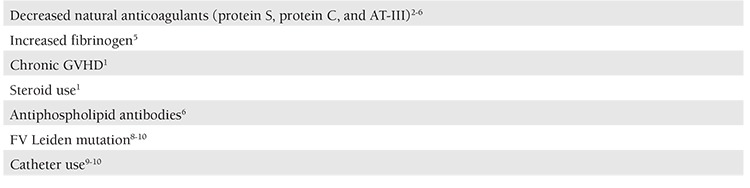
Reported risk factors for the development of DVT post-SCT.
